# Development of a closed‐artery catheter‐based myocardial infarction in pigs using sponge and lidocaine hydrochloride infusion to prevent irreversible ventricular fibrillation

**DOI:** 10.14814/phy2.12121

**Published:** 2014-08-28

**Authors:** Rafael Dariolli, Celso K. Takimura, Carlos A. Campos, Pedro A. Lemos, José E. Krieger

**Affiliations:** 1 Heart Institute (InCor), University of São Paulo Medical School, São Paulo, Brazil

**Keywords:** Balloon, catheter, embolism, fibrillation, myocardial infarction

## Abstract

The objectives of this study were to develop a robust, homogeneous, viable and inexpensive model of closed‐artery catheter‐based model of myocardial infarction (MI) in pigs without major cardiac dysfunction. Suitable animal models that mimic human cardiovascular conditions are of paramount importance to understand the effects of novel therapeutic strategies to improve tissue perfusion and prevent cardiac deterioration post‐MI. Pigs (*N* = 21, BW = 17 ± 1 kg) receiving continuous iv lidocaine hydrochloride were subjected to percutaneous intracoronary implant of foam sponge into the proximal left circumflex coronary artery. Intraprocedure mortality was 23.8%. ST segment elevation and increased serum Troponin T and CK‐MB were documented in all animals. Thirty days after occlusion, echocardiography (95% IC [9.3–12.4%]) and anatomopathological (95% CI [9.3–12.6%]) analyses confirmed a significant and reproducible MI. Taken together, we provide evidence for a suitable closed‐artery catheter‐based method to produce MI in pigs accompanied by tissue hypoperfusion and absence of overt heart failure.

## Abstract (precis)

We provide evidence that an inexpensive and easily available material can be used to produce a robust and homogenous percutaneous closed‐artery model of MI in pigs, when associated with lidocaine hydrochloride use. Thirty days after occlusion, anatomopathological (95% IC [9.3–12.6%]) analyses confirmed a significant and reproducible MI accompanied by hypoperfusion and absence of overt heart failure.

## Introduction

Despite great therapeutic progress, coronary artery disease (CAD) remains a challenging clinical problem WHO 2008. In the last decade, a number of cell/gene approaches have been developed with promising results in experimental studies, especially with rodents (Haider et al. 2004; Nakamuta et al. 2009; Danoviz et al. 2010; Gonçalves et al. 2010). Unlike anticipated, current evidence from experimental studies does not indicate that the beneficial effects of cell/gene therapy on cardiac function are linked to transdifferentiation or robust tissue replacement (Kinnaird et al. 2004; Wollert and Drexler 2005; Nakamuta et al. 2009; Danoviz et al. 2010). Instead, the effects appear to be largely related to paracrine action, through processes influencing the reduction in apoptotic cell death, regulation of inflammatory responses and neo‐angiovasculogenesis, which may find relevant clinical applications but it is unlikely to ameliorate overt cardiac failure states (Nakamuta et al. 2009; Danoviz et al. 2010; Mummery et al. 2010). This may explain, at least in part, inconsistencies observed between the promising early preclinical data obtained under controlled experimental conditions and large clinical trials designed to improve cardiac function or clinical outcomes (Dib et al. 2006; Makkar et al. 2012).

Suitable animal models, especially the ones that mimic cardiovascular structure and function in man, are of paramount importance to better understand the effects of novel strategies for myocardial reparation (Dondelinger et al. 1998; Hughes et al. 2001). In this context, a number of experimental models of myocardial infarction have been described in swine using percutaneous techniques (Eldar et al. 1994; Dib et al. 2006; Suzuki et al. 2008, 2009; Carlsson et al. 2009; Varga‐Szemes et al. 2013). However, most of these models induce myocardial injury by transitory ischemia (ischemia/reperfusion) (Suzuki et al. 2008; Buszman et al. 2012; Duran et al. 2012; Mazo et al. 2012), and not permanent coronary occlusion. Moreover, most of these models target occlusion of the anterior descending coronary artery (LAD) generating highly dysfunctional states (Carlsson et al. 2009). Also, the commonly used open‐artery models are not the most appropriate to explore some of the components of the regenerative mechanisms involved with cardiac function, such as neo‐angiovasculogenesis.

Closed‐artery models of myocardial infarction have been successfully obtained with materials that are expensive and not ready available (Eldar et al. 1994; Dib et al. 2006; Carlsson et al. 2009; Suzuki et al. 2009; Varga‐Szemes et al. 2013). In contrast, Reffelmann et al. (2004) described the use of foam sponge fragments as embolic particles, but their results displayed high mortality during the first week, mainly associated with arrhythmias followed by irreversible ventricular fibrillation even if the very small injury generated.

In this study, we extend the available models and describe a closed‐artery myocardial infarction procedure by occlusion of left circumflex coronary artery (LCx) that results in significant 9–12% myocardial infarction (MI) area of the Left Ventricle (LV) without major basal cardiac dysfunction suitable to investigate strategies to stimulate neo‐angiovasculogenesis. This is a closed chest, reproducible, homogeneous, inexpensive and followed up for 30 days model of infarction that minimizes experiment‐related early high mortality using easily available foam sponges and a continuous infusion of lidocaine hydrochloride to prevent ischemia‐related arrhythmias.

## Materials and methods

All experiments were conducted according to the protocol approved by the Institutional CAPPesq Ethics Committee (protocol number #022/09). The Animal care was complied based on the ARRIVE guideline (Animals in Research: Reporting *In Vivo* Experiments) (Kilkenny et al. 2010). Twenty‐four female pigs (*Sus scrofa domestica*, MS60 EMBRAPA lineage – weight, 15–20 kg) were maintained in a local commercial swine farm (Granja RG, Suzano‐SP, Brazil) with free access to food and water during the protocol. For acclimatization, the animals were brought to the experimental facility at least 24 h prior to the procedure.

Overnight fasted animals were sedated with a combination of ketamine chloridrate (8 mg/kg, im, Vetbrands, Jacareí, Brazil) and midazolam hydrochloride (0.5 mg/kg; Roche, Rio de Janeiro, Brazil). After 10–20 min, a cannula was introduced in a superficial ear vein. The anesthesia was induced intravenously with sodium thiopental (12.5 mg/kg; Cristalia, Sao Paulo, Brazil) and then orotracheal intubation (7‐ to 7.5‐mm tube – Chilecom, Guangdong, China) was performed. Anesthesia was maintained with isoflurane (1.5% to 2.5% – Baxter Health Care Corporation, Guayama, Puerto Rico – in 100% oxygen – in anesthetic equipment (Origami Ergo System – Takaoka). Before the procedure, the pigs received intramuscular benzathine penicillin 1.2 million units (Eurofarma, Sao Paulo, Brazil) to avoid infections. After the procedure, the pigs were treated with intramuscular sodium dipyrone (1000 mg, four times; two doses daily – Sanofi Aventis, Suzano, Brazil) to minimize pain.

The surviving animals underwent a new coronary angiography and transthoracic echocardiographic examination after 30 days, and then euthanized by an overdose of potassium chloride (30–40 mL of 19.1% solution, iv, Isofarma, Eusébio, Brazil) administrated under sodium thiopental deep anesthesia.

### Experimental design

The study was conducted in two periods. In a pilot phase, a group of three pigs underwent coronary occlusion as previously described by Reffelmann et al. (2004). Myocardial ischemia was induced by insertion of foam sponge fragments into the coronary lumen. Those animals comprised a control group that aimed to investigate the natural mortality after acute LCx occlusion.

In a second phase, a total of 21 swine underwent percutaneous coronary occlusion with foam sponge under continuous intravenous infusion of anti‐antiarrhythmic medications throughout the procedure.

### Percutaneous induction of myocardial infarction

Pigs under general anesthesia were fixed in supine position. After inguinal asepsis/antisepsis, a 6F vascular sheath (Merit Medical Systems, South Jordan, UT) was introduced in common femoral artery followed by administration of 10.000 IU of unfractionated heparin (Cristalia). The left coronary artery was selectively cannulated using a 6 French guiding‐catheter (Merit Medical Systems) under fluoroscopic guidance, followed by intracoronary injection of contrast (Pielograf 76% – Bracco Diagnostics Inc., Monroe Township, NJ) for baseline angiography acquisition. A 0.014” guidewire (BMH, Abbott Vascular Inc., Santa Clara, CA) was introduced to the distal portion of the LCx. Then, a small piece of a domestic foam sponge (Scotch‐Brite^™^, 3M do Brasil Ltda., Sao Paulo, Brazil; between 5 and 7 mm) was placed over the wire, and pushed to the proximal portion of LCx with the aid of a partially inflated balloon catheter (SeQuent^®^ – B. Braun Interventional Systems, Inc., Bethlehem, PA). An angiography was obtained 5–10 min after the sponge placement to confirm the mechanical obstruction of the vessel lumen. In case, a single sponge embolus was not sufficient to produce total coronary occlusion (thrombolysis in myocardial infarction – TIMI flow 0 or I), other fragments of sponge were introduced. After angiographic documentation of coronary occlusion, the balloon catheter was carefully positioned near the embolus to prevent movements of the sponges while the guidewire was removed from LCx. A final coronary angiography was performed to attest the persistent occlusion of the vessel, before the balloon and guiding catheter were removed. At the end of coronary procedures, the femoral artery was ligated and the incision was sutured. The animals were monitored and maintained on mechanical ventilation until the resumption of normal vital functions.

### Antiarrhythmic and electrical cardioversion procedure

To prevent episodes of malignant ventricular arrhythmias during LCx occlusion, a continuous intravenous infusion of lidocaine hydrochloride (1 mg/kg/h) (Cristalia) or amiodarone hydrochloride (1 to 1.5 mg/kg/h) (Sanofi‐Aventis) was administrated before, during and for at least 3 h after the myocardial infarction procedure. In case of ventricular arrhythmia episodes, extra bolus doses of lidocaine hydrochloride (2.5 to 12 mg/kg) or amidarone hydrochloride (1.0 to 3 mg/kg) were immediately administered as needed. If deterioration to ventricular fibrillation occurred, electrical defibrillation was performed (Codemaster XL – Hewlett‐Packard GmbH Medical Production, Boeblingen, Germany) by applying 200–300 J with the paddles pressed to the anterior chest wall, followed by close‐chest cardiac compression. The fibrillation was considered irreversible if refractory after 30 min.

### Diagnostic tests to cardiac biochemical markers

The measurements of circulating ultrasensitive troponin T and CK‐MB were performed in four representative pigs (four pigs for troponin T and two pigs for CK‐MB) according to the manufacturer's recommendations for the commercial kits (Roche). Serial serum measurements were performed immediately before and after LCx occlusion (every hour up to 24 h).

### Echocardiographic assessment

Fifteen animals underwent echocardiographic analysis using commercially available echocardiograph (Sonos 5500^®^; Philips Medical Systems, Andover, MA) 30 days after the MI and seven controls before MI to establish basal level values. Linear measurements were made of cardiac structures and flow. These measures were undertaken in order to quantify cardiovascular performance in these animals.

### Anatomopathological analyses

After euthanasia, the hearts were extracted and dissected to obtain only the LV, which was sectioned transversely from base to apex in sections of 5 mm (on average seven sections) and directed to specific stain techniques.

The section number 3 (middle of the infarcted area) was divided into three segments: remote, border zone and myocardial infarction itself. These segments were fixed in 4% paraformaldehyde. After 48 h of fixation, the tissues were placed in plastic cassettes and processed in a total cycle of 12 h for dehydration, diaphanization and paraffinization (Leica TP1020 – Leica Microsystems Inc., Buffalo Grove, IL). The paraffin‐embedded tissues were cut into microtome (4 *μ*m thick) and mounted on slides and stained with *Picrosirius Red* for measurement of interstitial collagen. Ten random images with 200X magnification of the injured and remote area of MI were obtained and quantified by automatic detection of colors using Leica QWin 3 software (Leica QWin Plus V 3.5.1 – Leica Microsystems Inc.). The results were expressed as percentage of positive tissue for *Picrosirius red* relative to myocardial area on slide.

The remaining LV sections were stained with 2, 3, 5 Triphenyl Tetrazolium Chloride Stain (TTC, Sigma‐Aldrich, St. Louis, MO) for macroscopical assessments. A solution of 1% TTC in phosphate buffer pH 7.4 was used. The cross sections were incubated in 200 mL of TTC, agitation and 37°C for 15–20 min. After staining, the sections were incubated in buffered 10% formaldehyde at room temperature for 10 min. The slices were washed in running water and then fixed in 10% formaldehyde solution. Boards were mounted with all the slices rebuilding the heart, with visions of both the apex to the base as well the base to the apex. The boards were photographed and the area of necrotic tissue was evaluated by planimetry using the software ImageJ (Schneider et al. 2012). Briefly, external area of LV (epicardial wall) and cavity area (endocardial wall, excluding papillary muscles and trabeculae) were measured. Cavity area was deducted from external area resulting in LV area. Injured areas were measured by freehand trace. The injured area values were deducted from LV area resulting in the noninjured LV area. The results were expressed by relative amount of all measured sections.

### Statistical analysis

Results are expressed as mean ± standard error of the mean (SEM). Unpaired nonparametric Student's *t*‐test was utilized to compare two groups of data and nonparametric one‐way ANOVA was used to compare three or more groups or time points. All statistical analyses were performed using GraphPad Prism 5.0 (GraphPad Software Inc., CA, USA). All tests were two‐tailed and *P* values < 0.05 were considered significant.

## Results

### Early and late animal mortality

In the pilot phase with three animals, we observed a 100% early mortality. Two animals died during the procedure and one pig suddenly died 24 h after the LCx occlusion, most likely associated with irreversible ventricular fibrillation (Table 1).

**Table 1.** Early and late Animal Mortality and VF incidence in closed‐artery model of MI

Period	Death (%)	VF rate (%)	Death after VF (%)
	Without LH (*n* = 3)		
Procedure	66.6 (*n *=**2)	66.6 (*n *=**2)	66.6 (*n* = 2)
Up to 24 h	33.3 (*n* = 1)	33.3 (*n* = 1)	33.3 (*n* = 1)
Up to 1 month	X	X	X
	With LH (*n* = 21)		
Procedure	23.8 (*n* = 5)	28.6 (*n* = 6)	19.0 (*n* = 4)^1^
Up to 24 h	X	X	X
Up to 1 month	4.8 (*n* = 1)	4.8 (*n* = 1)	4.8 (*n* = 1)^2^

LH, lidocaine hydrochloride solution; VF, ventricular fibrillation.

^1^ Or 57.0% of the 7 pigs that had VF.

^2^ Or 14.0% of the 7 pigs that had VF.

In the second experiment phase, where lidocaine hydrochloride solution was infused throughout and after the intervention, procedure‐related death was reduced to 23.8%, with only 5 of 21 pigs dying within first 24 h after LCx occlusion (Table 1). Subsequently, one pig (4.8%) died 15 days after the procedure (Table 1). Therefore, the lidocaine‐supported LCx occlusion resulted in 71.4% survival thorough the 30‐day period post‐MI (Table 1). During the procedure, 15 pigs required an extra‐dose of lidocaine in bolus. Six of these animals still had ventricular fibrillation and it were performed electrical cardioversion (Table 1). The use of amiodarone as antiarrhythmic as described failed to prevent irreversible ventricular fibrillation resulting in early death of all animals (*N* = 3) post‐LCx occlusion.

The animal losses in the second phase of the study were mostly related to technical problems during sponge positioning (Table 2). From the six deaths occurring during the whole 30‐day period, two pigs died because of coronary air embolism and two other animals died due to thrombotic occlusion of the left coronary artery. To overcome these technical issues, the kit of catheters was replaced to prevent coronary air embolism and the guidewire presoaked in saline to avoid direct contact between the dry‐guidewire and blood, a major cause of air bubbles formation. Second, the sponges were positioned close to the proximal to medial portion of LCx and the balloon catheter was only removed of proximal portion 5–10 min after confirming the occlusion to prevent sponge movements.

**Table 2.** Pigs mortality per cause in pigs treated with LH

Cause	Animals	Percentage
Pulmonary embolism	1	4.8^1^
Coronary air embolism	2	9.5
Left main occlusion	2	9.5
Late complications	1	4.8
Total	6	28.6

^1^ This animal did not show ventricular fibrillation followed by death.

### Procedure findings in the lidocaine‐supported study

**Figure 1.**
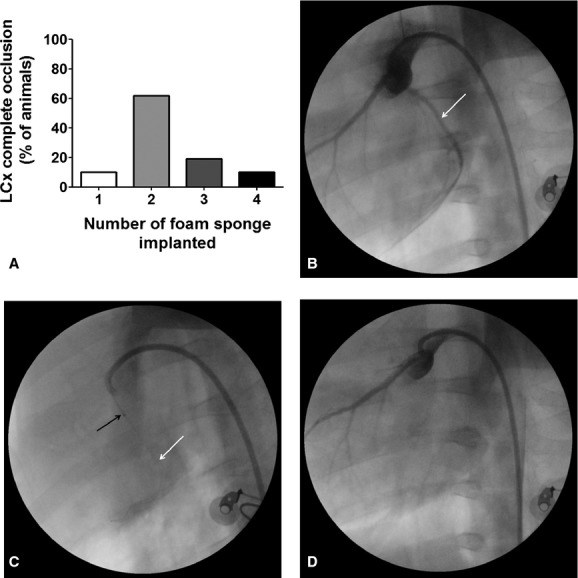
Foam sponge implanted in LCx. (A) Number of pieces of foam sponge to completely occluded the lumen of LCx. (B) First, a basal angiography was performed to localize the coronary tree. (C) after localized the target (proximal portion) a 0.014 guidewire (white arrow) was carried up to distal portion of LCx. Through the guidewire and assisted by a balloon catheter (black arrow) pieces of foam sponge were placed into LCx lumen. (D) Five to ten minutes after occlusion catheters and guidewire were removed from artery.

The average duration of the procedure was 54 ± 17 min in the second phase. Complete occlusion of LCx required one to four pieces of foam sponge (Fig. 1A). Five to ten minutes after positioning of the sponges in the proximal LCx, angiographic images confirmed the complete occlusion of the coronary from proximal portion (Fig. 1B–D). A decrease in mean blood pressure was commonly observed after LCx occlusion, whereas heart rate and pulse oxygen saturation remained unchanged (Table 3).

**Table 3.** Physiological parameters during MI induction in pigs treated with LH

	LCx occlusion	
	Before	After
Heart hate (bpm)	103 ± 16	100 ± 15
Systemic Pressure (mmHg)	72.0 ± 16	62.4 ± 17^1^
Pulse oxygen saturation	97.7 ± 1	98.1 ± 1

bpm, beats per minute.

^1^ *P* < 0.05 Systemic Pressure after x before LCx occlusion.

**Figure 2.**
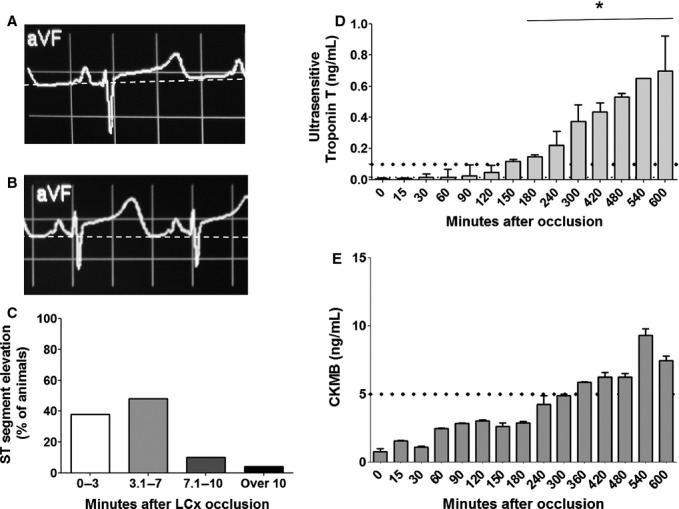
Diagnoses of ischemia and necrosis in pigs with occluded LCx. (A) ECG traces were recorded in each animal before LCx occlusion and used as basal traces (B) ECG traces showed important unlevelling of ST segment after occlusion suggesting beginning of ischemia. (C) Ninety‐six percent of the animals showed ST alternation between 2 and 10 min after LCx occlusion. To confirm the necrosis injury two animals had their blood sampled for about 10 h after LCx occlusion. (D) Ultrasensitive Troponin T dosage showed significant increase above reference value for MI 3 h after occlusion (ANOVA, *P < 0.05, time 0 vs other times, *n* = 4) (E) and CKMB showed a trend to be higher than reference value 6 h after occlusion (*n *= 2) corroborating Troponin T data.

Within 2–10 min after LCx occlusion, marked ST‐segment changes were observed in all animals (Fig. 2A–C). Four animals were randomly selected to serial blood sampling (from time 0 till 11 h post‐LCx occlusion) to assess serum necrosis biomarkers. Troponin T and in CK‐MB levels showed the expected time‐dependent increase (3 and 6 h, respectively), confirming the necrotic damage in cardiac tissue (Fig. 2D and E, respectively).

### Thirty‐day findings in the lidocaine‐supported study

**Figure 3.**
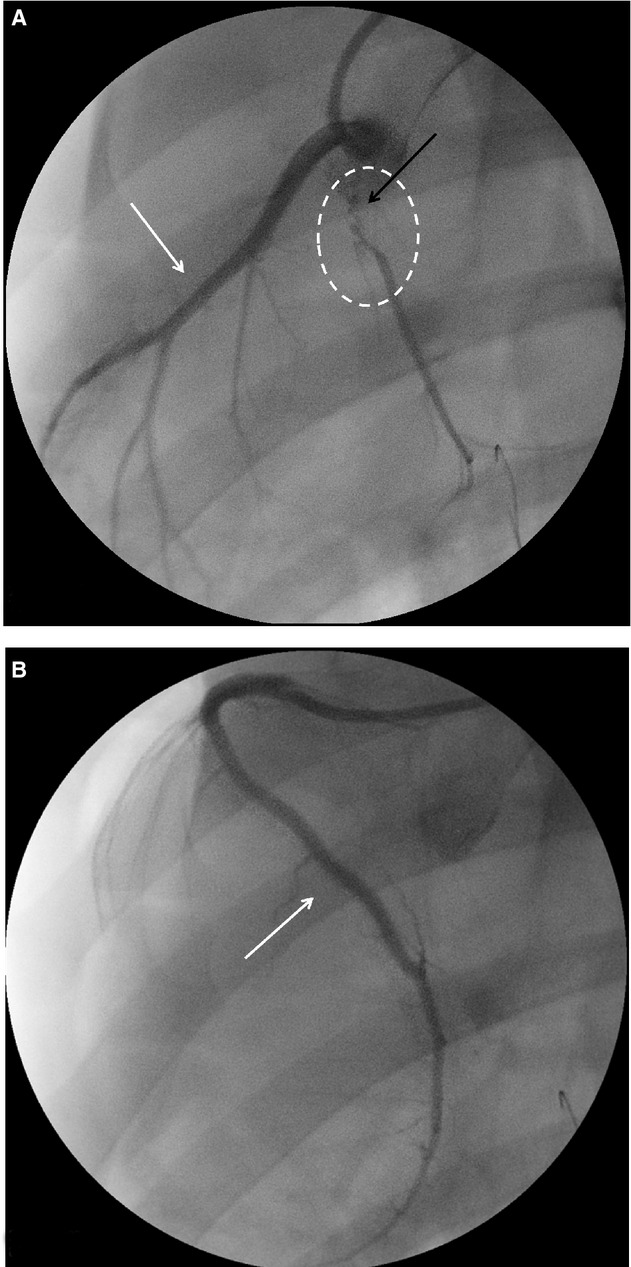
Scarce insufficient intracoronary collaterals were formed 30 days after LCx occlusion. Thirty days after occlusion angiographic images were performed to visualize coronary tree. (A) First, no migration of embolized sponges was observed (black arrow). (A and B) No irregularities or new occlusions were observed in left anterior descending coronary and right coronary arteries (white arrows). (B) Finally, only scarce and insufficient intracoronary collaterals were observed (white circle).

The surviving animals (*N* = 15) underwent angiographic evaluation 1 month after LCx occlusion. The embolized sponges did not migrate in any animal (Fig. 3A, white arrow). Nontarget vessels (i.e. right coronary artery and left anterior descending artery) were not occluded and did not show any abnormalities (Fig. 3A and B, white arrows). All animals showed only scarce intracoronary collaterals in LCx that generate insufficient blood‐flow revascularizations (Fig. 3A).

After 30 days, echocardiographic assessment showed hypokinesia or akinesia of posterior left ventricle wall in all infarcted animals. Furthermore, all pigs showed moderate to important mitral regurgitation (data not shown). LV shortening fraction (LVSF) and LV ejection fraction (LVEF), indicators of cardiac function, decreased at 30‐day post‐MI compared to baseline values (Table 4).

**Table 4.** Echocardiographic parameters 30 days after LCx occlusion in pigs treated with LH

	Healthy (*n* = 7)	MI (*n* = 15)
	Mean ± SD	Mean ± SD
Injured area (%)	x ± x	10.87 ± 2.73
Septum (cm)	0.41 ± 0.02	0.53 ± 0.08*
Posterior wall (cm)	0.44 ± 0.02	0.39 ± 0.07
EDD (cm)	3.33 ± 0.21	4.38 ± 0.32^§^
ESD (cm)	2.01 ± 0.17	3.50 ± 0.39^§^
LVSF (%)	39.55 ± 6.09	19.95 ± 7.43^‡^
EDV (cm)	45.41 ± 7.02	87.44 ± 14.44^‡^
ESV (cm)	13.00 ± 2.80	51.92 ± 13.13^‡^
LVEF (%)	70.97 ± 7.29	40.37 ± 13.00^†^
LV Mass (g)	30.89 ± 3.73	54.41 ± 8.42^‡^

EDD, end‐diastolic diameter; ESD, end‐systolic diameter; LVSF, left ventricular shortening fraction; EDV, end‐diastolic volume; ESV, end‐systolic volume; LVEF, left ventricular ejection fraction; LV Mass, left ventricular mass.

*P < 0.05; ^†^*P* < 0.005; ^‡^*P* < 0.0002, ^§^*P* < 0.0001; Student's *t*‐test MI vs. healthy animals.

**Figure 4.**
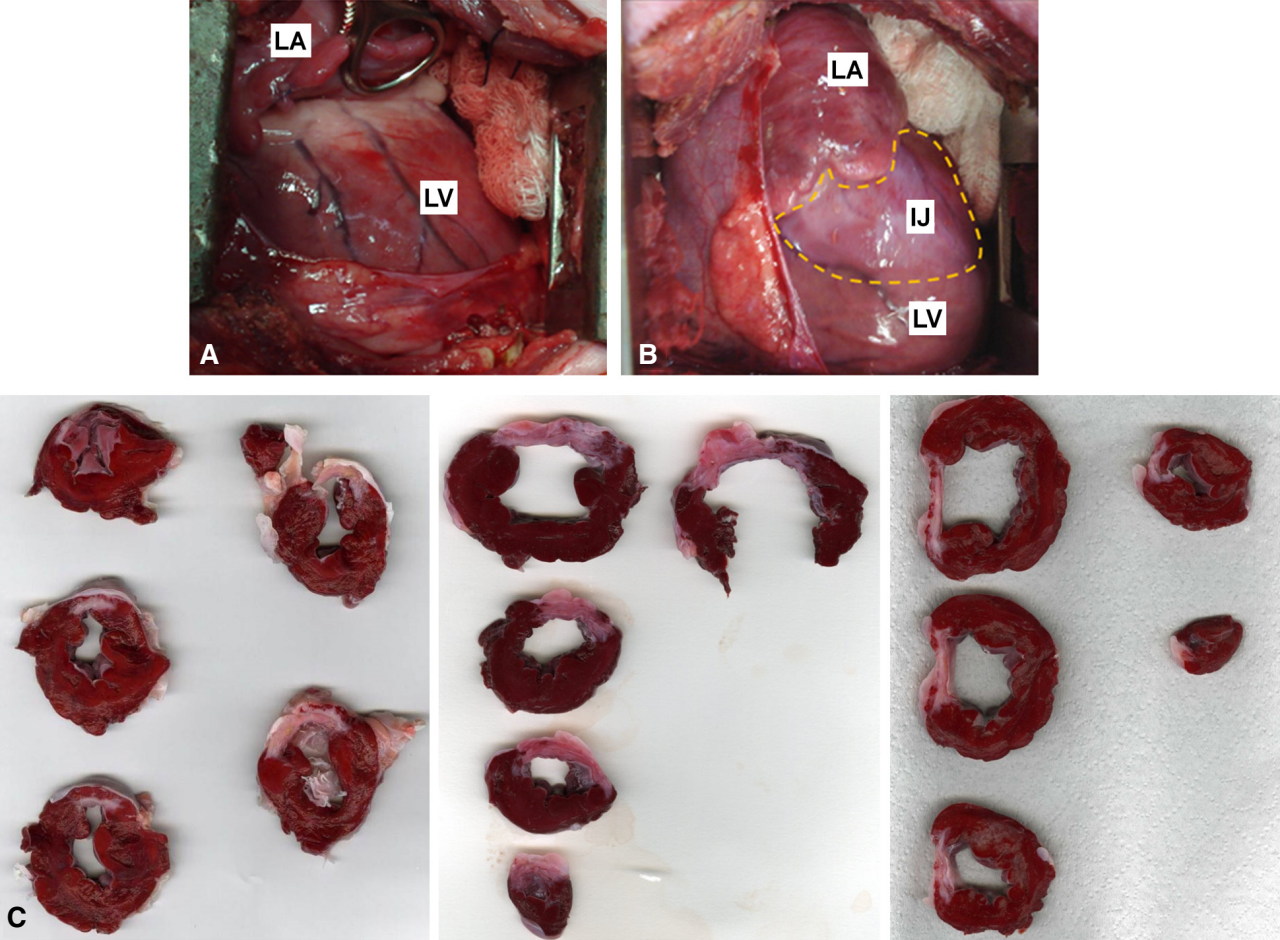
Anatomopathological assessment of LV 30 days after LCx occlusion. (A) Open‐chest view of LV posterior wall before LCx. (B) Open‐chest view of LV posterior wall 30 days after LCx occlusion. Note injured area in white (discontinuous yellow points). (C) Sliced sections of LV from three different pigs. Note that the injured areas are similar. LV, left ventricle; LA, left atrium; IJ, injured area.

**Figure 5.**
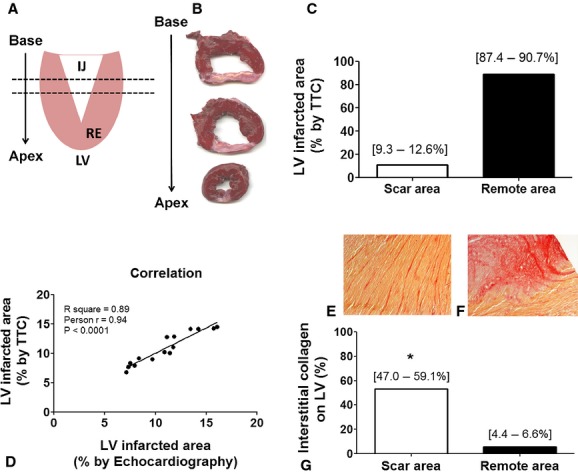
Anatomopathological follow‐up 30 days after LCx occlusion – histological confirmation and quantification of infarcted area. (A) After euthanasia the heath was isolated, LV dissected and sliced base to apex as schematic figure. (B) The injury extended from the base to the apex forming an inverted triangle clearly observed in LV slices stained with TTC. (C) LV injured perceptual area was homogeneous for all the animals (*n* = 15) and (D) showed strong correlation with echocardiographic measures (*n* = 15). Furthermore, a significant increase in interstitial collagen in (E) remote and (F) injured areas were (G) quantified versus the amount of collagen in healthy animals LV (2% of interstitial collagen all over the LV;* P* < 0.001). IJ, injured area; RE, remote area.

**Figure 6.**
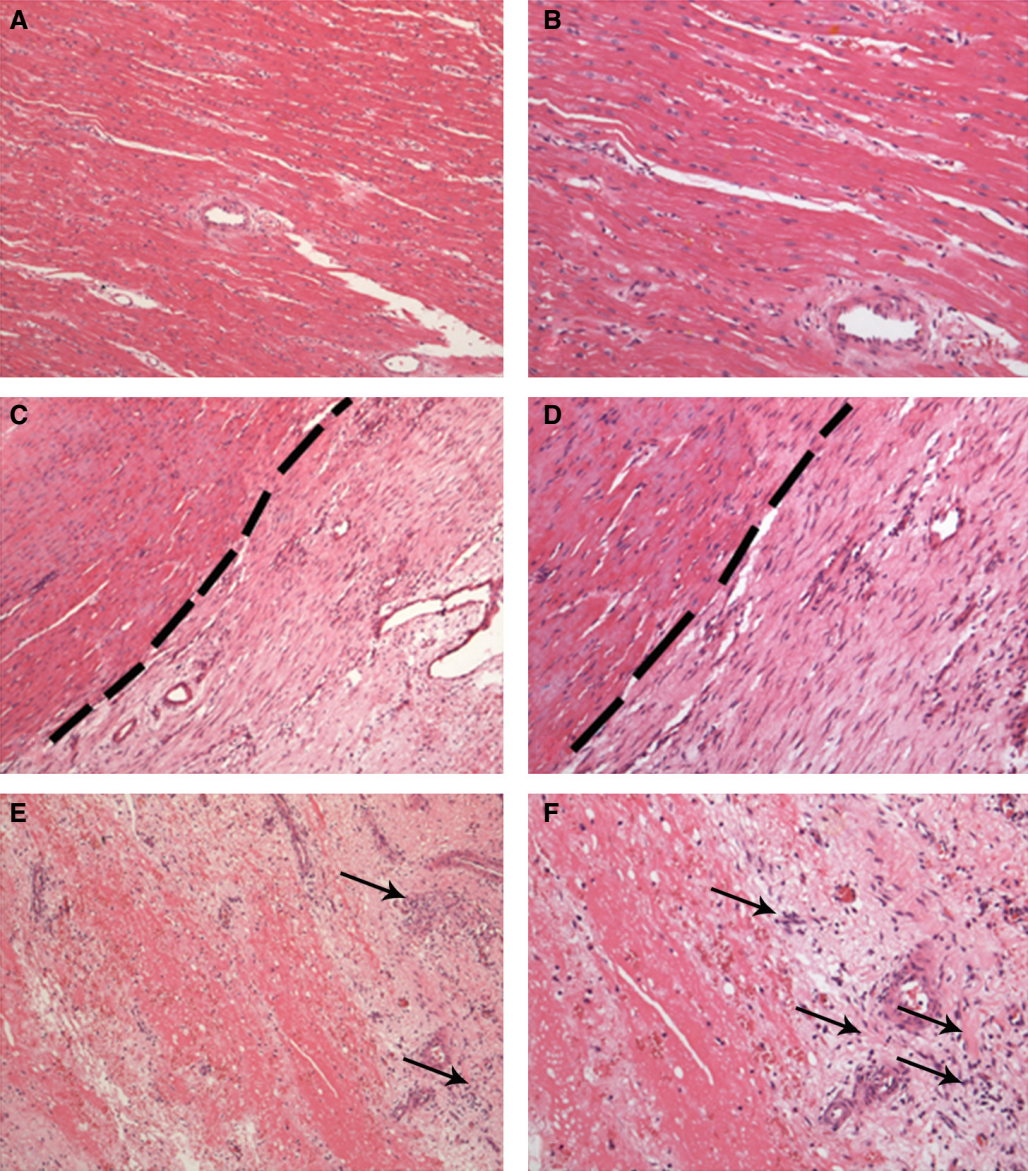
Histological diagnosis of MI 30 days after LCx occlusion. (A and B) Longitudinal section of remote area of MI (septum wall). Note organization of the cardiac muscle fibers. (C and D) Longitudinal section of border area of MI. Note that in dashed line there is clear differences in organization and composition of cardiac tissue. (E and F) Longitudinal section injured area (posterior wall). Note the complete disruption of the cardiac muscle fibers and inflammatory infiltrated cells (black arrows). All sections were stained with H&E (A, C and E – 100 × magnification, and B, D and F – 200 × magnification), *n *=**15.

The anatomopathological assessment showed an injury pattern that was similar in all excised hearts (Fig. 4). The injury extended from the base to the apex of the heart forming an inverted triangle (Fig. 5A and B). On average, the myocardial infarction area was 10.95 ± 2.99% (95% IC of mean [9.3–12.6%]) of the left ventricle (Fig. 5C). These data showed significant correlation with echocardiographic measurement of MI area (10.87 ± 2.73% with 95% IC of mean [9.3–12.4%]; Fig. 5D). There was also a significant increase in interstitial collagen in the remote and injured areas (Fig. 5E–G) while in healthy animals this value did not exceed 2% in the entire length of the left ventricle. Furthermore, histological slices stained with hematoxylin and eosin showed the classic pattern of the muscle fibers disorganization and collagen deposition in MI and border zone (Fig. 6).

## Discussion

In this study, we provide evidence that an inexpensive and easily available material can be used to produce a robust and homogenous percutaneous closed‐artery model of MI in pigs, when associated with lidocaine hydrochloride use.

Although limited, due to the high mortality, the data first described by Reffelmann et al. (2004) has been successfully extended and improved to achieve a low mortality and homogenous model of myocardial infarct in pigs.

Even today, a wide range of studies still employ surgical, open‐chest, models of MI in large animals such as pigs (Tuzun et al. 2010; Weiss and Saint 2010; Cheng et al. 2011). These models impose intrinsic acute and postoperative difficulties (Munz et al. 2011; Lukács et al. 2012) due to their invasive nature, which ultimately result in high mortality rate and elevated costs, or adversely affect the homogeneity of the injury among the animals. *Munz and colleges* showed 67% of mortality in an LCx surgical model of MI in pigs (Munz et al. 2011), whereas the same parameter was less than 30% in our proposed model.

Previously reported percutaneous models of ischemia/reperfusion have been described, using balloon catheters to temporarily occlude the vessel lumen, as well as, agarose gel microbeads (Eldar et al. 1994), and apatite‐coated bioabsorbable polymer sponge (Suzuki et al. 2009). Differently, closed‐artery models make use of microembolization with Embospheres (Carlsson et al. 2009; Varga‐Szemes et al. 2013), coil deployment (Dib et al. 2006), foam sponge (Reffelmann et al. 2004), among other to obtain permanent occlusion of coronary arteries. Closed‐artery models of MI maintain a chronic low‐flow status at the injured area and, therefore, are better suited for studies aiming to explore the processes associated with neo‐angiovasculogenesis, which is believed to be critical in preventing cardiac deterioration (or even improving cardiac function) post injury (Nakamuta et al. 2009; Danoviz et al. 2010; Gonçalves et al. 2010).

The LCx occlusion model resulted in 9–12% scar formation in the LV and decrease (LVSF, 20% vs. 40% and LVEF, 40% vs. 71% compared to healthy pigs), albeit these findings are less important than the ones reported to models of LAD occlusion associated with overt impairment of cardiac function (Carlsson et al. 2009; McCall et al. 2012). Thus, considering that effective means to replace lost cardiomyocytes are yet to be developed, the current strategies should be tested for their role in improving or preventing further cardiac deterioration associated with cardiac tissue ischemia and not reversing overt heart failure states.

Domestic foam sponges are inexpensive and readily available, as well as easy to sterilize and to manipulate during the percutaneous closure of the artery. The first report describing the model attempted to occlude only the distal third of LCx, but showed high a mortality rate (52% at 7 days) (Reffelmann et al. 2004) differently of our model that provide larger occluded portion of LCx (proximal occlusion) with a better survival in a greater follow‐up (viability of 71.4% at 30 days). In *Reffelmann*'s report the losses were mainly associated with arrhythmic episodes (Reffelmann et al. 2004). Similarly, in our first attempts to occlude LCx, all the animals showed arrhythmic episodes followed by irreversible ventricular fibrillation.

With the objective of decreasing acute arrhythmic death related to the coronary occlusion, we first tested the use of intravenous amiodarone with little success. This is consistent with previous reports showing that intravenous amiodarone may not take effect immediately after the start of infusion in pigs (Tsagalou et al. 2004). We then used lidocaine hydrochloride, which had been successfully used for ventricular fibrillation in pigs (Reynolds et al. 2007). The experimental protocol was thoughtfully planned to include continuous lidocaine infusion before, during and after the occlusion of LCx to minimize arrhythmias (mainly 35–45 min after occlusion), together with allowing for extra lidocaine boluses if needed. It is important to emphasize that the use of lidocaine did not affect the reproducibility of the myocardial injury induced by LCx occlusion, which produced a highly homogenous infarcted are among the individuals.

It is important to emphasize that presently we lack effective means to replace large number of cardiomyocytes losses that may occur following large myocardial infarction so overt heart failure cannot be targeted yet. In contrast, gene/cell approaches explored in the last 10 years have provided evidence that local inflammation, deposition of collagen and cell death can be targeted and may have clinic relevance to specific human ischemic conditions. Thus, there is need in the field to careful validate and optimize these early successful interventions in larger animals such as the swine model proposed here. In this type of model clear ischemia without overt cardiac dysfunction will be useful address important issues regarding timing and routes of intervention, dose response and association with other treatments, to name a few, to assess the rapid translation of these approaches into clinical settings.

## Conclusion

Altogether, we provide evidence for a robust, homogeneous and viable model of percutaneous LCx closed‐artery model of MI in pigs. The use of domestic foam sponge to permanently occlude the artery and lidocaine hydrochloride infusion before, during and after the occlusion procedures rendered a significant MI with low mortality rate in pigs.

## Acknowledgments

The authors want to thank Leonora Loppnow, Euclydes Fontegno Marques and Luís Felipe Neves dos Santos for their assistance during the development of catheter‐based model of MI in pigs.

## Conflicts of interest

The authors declare no conflict of interest. The funders had no role in study design, data collection and analysis, decision to publish, or preparation of the manuscript.
